# Impact of mRNA-based vaccines in the prevention of adverse outcomes of COVID-19 infection in pregnancy: a single-center cohort study

**DOI:** 10.3389/fped.2023.1214768

**Published:** 2023-10-24

**Authors:** Riccardo Tudisco, Cristina Garufi, Francesca Rizzo, Teresa Polimeno, Antonio Lanzone, Sara De Carolis

**Affiliations:** ^1^ Dipartimento Scienze Della Salute Della Donna, Del Bambino e di Sanità Pubblica, Fondazione Policlinico Universitario A. Gemelli IRCCS, Rome, Italy; ^2^ Arthritis Center, Reumatologia, Dipartimento di Scienze Cliniche Internistiche, Anestesiologiche e Cardiovascolari, Sapienza Università di Roma, Rome, Italy

**Keywords:** COVID-19 vaccination, pregnancy outcome, COVID-19 infection, COVID-19 pneumonia, birthweight percentile

## Abstract

Several data have suggested that pregnant women have an increased risk of severe COVID-19 compared to those who are not pregnant. Moreover, different studies have showed that severe COVID-19 is limited mostly to unvaccinated women. The aim of the present study was to ascertain the different maternal and fetal outcomes in pregnant women with COVID-19 according to their vaccination status. A retrospective cohort study was carried out including all women admitted to the high-risk pregnancy unit of our center with COVID-19 between December 2021 and February 2022. Among the 163 women included in the study, 60 were vaccinated with an mRNA vaccine and 103 were unvaccinated. Pregnancy outcome and obstetrical and neonatal complications were encountered. Vaccinated women showed higher educational levels and lower prevalence of cases, with BMI >25 compared to unvaccinated women. Moreover, vaccinated women were admitted mostly for obstetrical indications rather than for COVID-related symptoms. In addition, the risk of developing COVID-19 pneumonia was significantly higher in unvaccinated women (*p* = 0.01) compared with vaccinated ones. Furthermore, pregnancy and neonatal outcomes showed some differences in the two cohorts. In unvaccinated women, the rate of C-section was higher (*p* = 0.03), and the mean birthweight percentile in their infants was impaired by COVID-19 infection (*p* = 0.01) when compared to those born to vaccinated women. Based on these results, we suggest that women who received a full course of vaccination were protected from the severity of the disease, having milder symptoms of SARS-Cov2 infection, while also presenting a more favorable pregnancy outcome.

## 1. Introduction

Coronavirus disease 2019 (COVID-19), caused by SARS-CoV2, was declared a global pandemic in March 2020. Observational data and meta-analysis have suggested that pregnant women have an increased risk of severe COVID-19 compared to those who are not pregnant. In fact, several countries have considered pregnant and postpartum women as an “at risk” priority group for COVID vaccination (1, 2).

As shown in a recent article, there is an increased risk of SARS-CoV-2 infection, hospitalization, and admission in intensive care units in unvaccinated patients, and vaccination is effective in reducing the risk of stillbirth, preterm birth, and neonatal ICU admission (3). Vaccination could be important for pregnant women and the general population to prevent the long-term sequelae of sarsCOV2 infection, known as “long COVID”. This includes neurodegenerative complications, dementia, and Parkinson's disease (4); neurological, physical, and psychological sequelae such as fatigue, sleep difficulties, impaired diffusion capacity for carbon monoxide, hair loss, dyspnea, and anxiety (5); and cardiac impairment in patients with a history of ICU admission (6).

Vaccination represents the most effective prevention strategy to contain the spread of infection and decrease the probability of severe disease. At the beginning of the vaccination period, pregnant women were excluded from clinical trials of COVID-19 vaccines and medication. Subsequently, observational data about the safety of mRNA-based vaccines for COVID-19 in pregnant women were reassuring, and their benefits have been documented by large studies (7–11).

The passage of anti-SARS-CoV-2 immunoglobulins through the placenta and breastmilk after COVID vaccination has been demonstrated in several studies (12–15).

Many obstetric societies recommend the adherence of all pregnant women to vaccination, but vaccine hesitancy is a widely spread phenomenon among pregnant women. Meanwhile, recent data have shown that severe COVID-19 is almost limited to unvaccinated women (16) and that unvaccinated pregnant women have a higher risk of contracting the COVID-19 virus (17).

The rate of COVID-19-associated ICU admissions among pregnant women increased threefold in Italy between February 2021 and June 2021 compared to February 2020 and January 2021 (18). Based on these findings and the mounting evidence of vaccine safety, Italy extended its vaccine recommendation to all pregnant women in September 2021 (circular letter from the Ministry of Health, 24 September 2021).

The aim of the present study is to verify whether different maternal and fetal outcomes occurred between vaccinated and unvaccinated pregnant women who were admitted to our high-risk pregnancy unit after the vaccine recommendation (December 1, 2021 to February 15, 2022) and resulted positive for COVID-19 infection.

## 2. Materials and methods

### 2.1. Study design and patients

We conducted a retrospective cohort study including all women who were admitted in the high-risk pregnancy unit of IRCSS, Fondazione Policlinico Gemelli of Rome, between December 1, 2021 and February 15, 2022. Patients with twin pregnancies or patients with unknown vaccination status were excluded from enrolment. Twin pregnancies were excluded because multiple gestations “per se” are more prone to obstetrical complications, and the indicators of pregnancy outcomes (week of gestation, birthweight, and birth percentile) are not comparable to those of singleton pregnancies. Patients' medical records of maternal and neonatal characteristics along with vaccination status were recorded.

### 2.2. Variables definitions

The clinical variables included in the analysis were maternal age, level of education (tertiary education), maternal weight, pre-gestational body mass index, presence of known thrombophilia, presence of pregnancy-related complications, length of hospitalization, route of delivery, gestational age at delivery, birth weight, birth weight percentile according to Ferrazzani et al. (19), number of infants small for gestational age (defined as birthweight <10th percentile), and APGAR score at 1 min and at 5 min.

### 2.3. Indicators of adverse outcomes

The indicators of outcomes were live births, spontaneous abortion, fetal loss, intrauterine death, neonatal death, small for gestational age (SGA), preterm delivery, and rate of caesarean sections. Moreover, the week of delivery, birth weight, and birth weight percentile were encountered.

### 2.4. Pathology

Histological changes in the placenta were evaluated through pathological examination. Placental findings were reported according to the synoptic framework proposed by Benton et al. (20).

### 2.5. Statistical analysis

Descriptive statistics included the observed number of cases for categorical variables, while quantitative variables were expressed as mean and standard deviation. Categorical variables were compared using the *χ*^2^ test and Fisher's exact test. *T*-test was used to compare the distribution of continuous data between the two groups. The statistical analysis was conducted at a 95% level of confidence and a 5% level of statistical significance.

## 3. Results

### 3.1. Population characteristics

Out of the total of 175 women admitted during the study period, 163 were eligible and enrolled in the study (60 vaccinated and 103 unvaccinated women). Patients suffered from the wild-type, Omicron, and Delta variants of COVID-19. Omicron was the most represented variant at the time of the study in Europe (21, 22) and in Italy (according to report no. 17 of 18th February 2022 of the Italian National Institute of Health) (23).

In regard to the number of doses received, it was observed that 47 (78,3%) received two doses of the mRNA-based vaccine in the second and third trimesters, while 13 patients received only one dose during the second and third trimesters because they suffered from previous COVID-19 infection before pregnancy (*n* = 5) or received two doses before pregnancy (*n* = 8).

The background characteristics of the two cohorts are shown in Table 1. Compared to unvaccinated women, vaccinated ones presented a significantly lower rate of overweight/obesity. Patients with tertiary education were more diffuse in the cohort of vaccinated women.

**Table 1:** Comparison of background characteristics according to vaccination status.

	Unvaccinated (*n* 103)	Vaccinated (*n* 60)	*P*-value
Maternal background characteristics			
Maternal age, years (mean ± SD)	33.79 ± 7.66	33.53 ± 4.67	0.37
Pregestational BMI^a^, kg/m^2^ (mean ± SD)	24.45 ± 4.97	23.97 ± 4.14	0.26
Number of patients with BMI > 25	35	22	**0**.**04**
Number of patients with tertiary education	22	19	**0**.**04**
Cause of admission to the hospital:			
- Obstetrical	87	58	**0**.**03**
- Covid-related	16	2	
Severity of COVID-19 symptoms:			
- Asymptomatic or pauci-symptomatic	90	59	**0**.**01**
- COVID-19 pneumonia	13	1	
Length of maternal hospitalization, days (mean, range)	6.22 (2–26)	5.05 (3–13)	**0**.**02**
Pregnancy comorbidities			
Thrombophilia	7	4	0.97
Gestational diabetes	14	8	0.96
Pregnancy-related hypertensive disorders	6	1	0.21

^a^ BMI, body mass index.

The bold values indicated the statistically significant *p* values (*p* < 0.05).

Patients who received a full course of vaccination had a lower risk of admission for COVID-related symptoms; in fact, they were admitted mostly for obstetrical reasons (*p* = 0.01). Moreover, the risk of developing COVID pneumonia with the necessity of oxygen supplementation was higher in unvaccinated patients [OR 8.52 95% CI (1.08–66.8864), *p* = 0.04]. In our cohort, none of the vaccinated women were admitted in the ICU or needed high-flow oxygen therapy, while 7 of the 103 unvaccinated patients required such support. In addition, patients who did not receive the course of vaccination had a significantly longer period of hospitalization, with the longest hospitalization reaching 26 days.

### 3.2. Delivery and postpartum outcomes

Of the 163 patients admitted in our unit, 146 delivered during the period of admission. In Table 2, we report the pregnancy outcome, mode of delivery, and neonatal characteristics of the unvaccinated and vaccinated women. We observed a higher rate of C-sections (*p* = 0.03) in unvaccinated patients who delivered during hospitalization.

**Table 2:** Comparison of delivery data and neonatal characteristics in the two groups of pregnant patients.

	Unvaccinated (*n* 94)	Vaccinated (*n* 52)	*P*-value
Delivery and postpartum characteristics			
Gestational age at delivery, weeks (mean ± SD)	38.32 ± 2.39	38.38 ± 2.28	0.44
Route of delivery:			
- Vaginal	56	40	**0**.**03**
- C-section	38	12	
Preterm Delivery			
- < 37 weeks	12	5	0.56
- < 34 weeks	2	3	0,35
- < 32 weeks	2	2	0.61
- < 28 weeks	0	0	–
Type of labor:			
- No labor	28	10	0.25
- Induction	32	24	
- Spontaneous	34	18	
Days of hospitalization	6.13 (2–26)	5.05 (3–13)	**0**.**02**
Neonatal characteristics			
Apgar < 7 at 5 min	0	0	–
Birthweight, grams (mean ± SD)	3,095.16 ± 24.84	3,199.60 ± 565.80	0.08
Birthweight percentile	51 ± 24.84	58.09 ± 24.80	**0**.**01**
Number of SGA^a^	5	1	0.32

^a^ SGA, small for gestational age, defined as birthweight < 10 percentile.

Wks, Weeks of gestation.

The bold values indicated the statistically significant *p* values (*p* < 0.05).

The infants of the unvaccinated women showed a trend of lower birthweight when compared to those of the unvaccinated women (*p* = 0.08), and the birthweight percentile was significantly reduced in infants born to the unvaccinated patients (*p* = 0.01). No difference was found in the two groups for the following features: preterm delivery (at <37 weeks, <34 weeks, <32 weeks, and <28 weeks), APGAR at 5 min, and gestational age at delivery.

Linear regression was used to determine the association of maternal characteristics (age, pregestational BMI, vaccination status) and pregnancy characteristics (preeclampsia, gestational diabetes) with the birthweight percentile. The analysis showed the correlation of neonatal sex (*R* = −8,737, *p* = 0.04) and preeclampsia (*R* = −41.18, *p* = 0.016). Moreover, the independent association between vaccination and the birthweight percentile was confirmed (*R* = 9.08, *p* = 0,04). No significant correlations were found between maternal age and pregestational BMI.

### 3.3. Pathological characteristics of placentas

Of the 146 patients who delivered during the period of admission, it was possible to retrieve 124 histological reports. Pathological lesions were reported according to the framework proposed by Benton et al. (20). In Table 3, we report the main pathological characteristics of placentas from the vaccinated and unvaccinated women. Placentas from the newborns of vaccinated mothers showed a lower rate of avascular fibrotic villi (*p* = 0.049) and intervillous thrombi (*p* = 0.01). No statistically significant differences were found in the two groups for the other pathological outcomes.

**Table 3:** Comparison of pathological characteristics of the placenta in the two groups of patients.

	Unvaccinated (*n* 80)	Vaccinated (*n* 44)	*P*-value
Weight, g (mean ± SD)	486,8 ± 120,09	487,8 ± 112,3	0,49
Category 1: Evidence of maternal vascular malperfusion			
Placental infarct(s)	5	2	0,52
Accelerated villous maturation pattern	5	3	0,58
Distal villous hypoplasia	16	7	0,38
Villous agglutination	60	29	0,19
Increased syncytial knots	76	41	0,48
Category 2: Evidence of maternal decidual arteriopathy			
Insufficient vessel remodeling	0	0	–
Fibrinoid necrosis	0	0	–
Category 3: Implantation site abnormalities			
Microscopic accreta	0	0	–
Category 4: Evidence of ascending intrauterine infection			
Inflammatory lesions	7	2	0,31
Category 5 Evidence of placenta villous maldevelopment			
Chorangiosis	21	11	0,53
Delayed villous maturation	6	3	0,60
Chorangiomas	1	0	0,64
Category 6: Evidence of fetal vascular malperfusion			
Avascular fibrotic villi	10	1	**0,049**
Intramural fibrin deposition	0	1	0,35
Stem villous vascular obliteration	10	6	0,53
Thrombosis	0	0	–
Villous stromal vascular karyorrhexis	3	0	0,26
Category 7: Fibrinoid			
Increased focal perivillous fibrin deposition	16	6	0,26
Category 8: Intervillous thrombi			
Intervillous thrombi	9	0	**0,01**
Category 9: Evidence of chronic inflammation			
Chronic inflammation	0	1	0,35

The bold values indicated the statistically significant *p* values (*p* < 0.05).

## 4. Discussion

We have confirmed that infection with coronavirus SARS-CoV2 in pregnant women confers an increased risk of ICU admission compared to non-pregnant women, as also demonstrated in recent literature (24, 25). Furthermore, we confirm the presence of COVID-19 vaccination barriers in our population despite Italy having extended its vaccine recommendation to all pregnant women, considering that only one-third of the enrolled population had the vaccination. A difference in educational status between the unvaccinated and vaccinated patients was also observed, in agreement with previous studies (26, 27).

Although our cohort represents only a small subset of patients from a single-center experience, including different variants of sarsCov2 (wild type, Omicron, and Delta variant), we observed a significant influence of COVID-19 in infants born to unvaccinated mothers, particularly for the mean birthweight percentile. In the literature, patients with COVID-19 are reported to present a higher risk of preeclampsia, stillbirth, and preterm birth (28). It is probable that the mechanisms underlying lower birthweight are the same as those that lead to preeclampsia and stillbirth. A pathogenic role of SARS-CoV-2 has been hypothesized through the binding to angiotensin convertin enzyme 2 receptors causing a renin–angiotensin system dysfunction and vasoconstriction (29), thus determining vascular damage of the placenta. Other studies suggest the possibility of an endothelial dysfunction induced by the proinflammatory state caused by SARS-CoV2 infection (30, 31).

Recently, it was reported that maternal SARS-Cov2 infection during pregnancy may increase the risk of an exacerbated thrombophilic status (32–35). In our series, we did not observe any case of clinically relevant maternal or neonatal thrombosis, probably because of the small size of the sample or because the omicron variant that was more diffuse at the time of the study has less aggressive behavior (22). In the present study, however, we confirmed the fact that the infection with COVID-19 led to a thrombophilic status, as evident from the histological examination of the placentas that showed a higher risk of histological thrombosis.

To our knowledge, different studies have shown differing pregnancy outcomes and maternal and fetal complications between vaccinated and unvaccinated patients (10). Based on the results of the present study, we can suggest that women who received a full course of vaccination were protected from the severity of the disease, experiencing decreased effects of coronavirus SARS-Cov2 infection, and had a more favorable pregnancy outcome.

More data are needed to explore the long-term safety of COVID-19 vaccination during pregnancy in respect to maternal and neonatal health.

We are aware that this study has some limitations due to its retrospective nature and the small group of patients enrolled in a single-center experience. However, the present results confirm the efficacy and safety of COVID-19 vaccination in pregnancy and suggest a potential role in protecting maternal health and fetal growth from the consequences of COVID-19 infection during pregnancy.

## Data Availability

The raw data supporting the conclusions of this article will be made available by the authors, without undue reservation.
